# Specific fatty acid intake and the risk of pancreatic cancer in Canada

**DOI:** 10.1038/sj.bjc.6602380

**Published:** 2005-02-01

**Authors:** A Nkondjock, D Krewski, K C Johnson, P Ghadirian, and the Canadian Cancer Registries Epidemiology Research Group

**Affiliations:** 1 Epidemiology Research Unit, Research Centre, Centre hospitalier de l'Université de Montréal (CHUM)-Hôtel-Dieu, Montreal, QC, Canada; 2 McLaughlin Centre for Population Health Risk Assessment, Institute of Population Health, University of Ottawa, Ottawa, ON, Canada; 3 Surveillance and Risk Assessment Division, Centre for Chronic Disease Prevention and Control, Population and Public Health Branch, Health Canada, Ottawa, Canada

**Keywords:** pancreatic cancer, fatty acid, stearate, oleate, fat, food frequency questionnaire, diet, cancer prevention

## Abstract

The possible association of specific fatty acid (FA) intake and pancreatic cancer risk was investigated in a population-based case–control study of 462 histologically confirmed cases and 4721 frequency-matched controls in eight Canadian provinces between 1994 and 1997. Dietary intake was assessed by means of a self-administered food frequency questionnaire. Unconditional logistic regression was used to assess associations between dietary FAs and pancreatic cancer risk. After adjustment for age, province, body mass index, smoking, educational attainment, fat and total energy intake, statistically significant inverse associations were observed between pancreatic cancer risk and palmitate (odds ratios (ORs)=0.73; 95% confidence intervals (CIs) 0.56–0.96; *P*-trend=0.02), stearate (OR=0.70; 95% CI 0.51–0.94; *P*-trend=0.04), oleate (OR=0.75; 95% CI 0.55–1.02; *P*-trend=0.04), saturated FAs (OR=0.67; 95% CI 0.50–0.91; *P*-trend=0.01), and monounsaturated FAs (OR=0.72; 95% CI 0.53–0.98; *P*-trend=0.02), when comparing the highest quartile of intake to the lowest. Significant interactions were detected between body mass index and both saturated and monounsaturated FAs, with a markedly reduced risk associated with intake of stearate (OR=0.36; 95% CI 0.18–0.70; *P*-trend=0.001), oleate (OR=0.36; 95% CI 0.19–0.72; *P*-trend=0.002), saturated FAs (OR=0.35; 95% CI 0.18–0.67; *P*-trend=0.002), and monounsaturated FAs (OR=0.32; 95% CI 0.16–0.63; *P*-trend<0.0001) among subjects who are obese. The results suggest that substituting polyunsaturated FAs with saturated or monounsaturated FAs may reduce pancreatic cancer risk, independently of total energy intake, particularly among obese subjects.

Pancreatic cancer represents the fourth leading cause of cancer-related deaths in Canada (CCS, 2004), and is among the most rapidly fatal cancer worldwide (WHO/IARC, 2003). The prognosis of subjects with pancreatic cancer is extremely poor since diagnosis is usually late due to lack of symptoms. The median survival time, regardless of therapy, is about 2–3 months after diagnosis, with a 5-year survival of about 4% (Ries *et al*, 2002).

Experimental studies of pancreatic cancer indicate that while dietary fat *per se* does not cause pancreatic cancer (Roebuck, 1992), individual polyunsaturated fatty acids (FAs), but not monounsaturated or saturated FAs, have an inhibitory effect on the growth of human pancreatic cancer cell lines (Falconer *et al*, 1994), suggesting that an association between dietary fats and pancreatic cancer may depend on the level of specific FA intake.

An elevated risk of pancreatic cancer in relation to high dietary total fat (Durbec *et al*, 1983; Ghadirian *et al*, 1991; Howe and Burch, 1996) and saturated fat intake (Ghadirian *et al*, 1991; Howe and Burch, 1996; Stolzenberg-Solomon *et al*, 2002) has been reported. Although no association with total or saturated fat intake has been found (Farrow and Davis, 1990; Kalapothaki *et al*, 1993), a significant decrease in risk with higher saturated (Bueno de Mesquita *et al*, 1990, 1991; Zatonski *et al*, 1991; Ji *et al*, 1995; Silverman *et al*, 1998), monounsaturated (Bueno de Mesquita *et al*, 1990; Zatonski *et al*, 1991), and polyunsaturated (Zatonski *et al*, 1991) fat intake has been reported. A recent cohort study found no significant association in relation to specific FAs (Michaud *et al*, 2003), but included less than 180 cases over 18 years of follow-up. The present case–control study, based on data from the Canadian National Enhanced Cancer Surveillance System (NECSS), examined whether the intake of specific FAs was associated with pancreatic cancer risk.

## METHODS

### Study population

The NECSS is a multi-site, population-based, case–control study involving 21 022 participants with one of 19 types of cancer identified through cancer registries in eight of the 10 Canadian provinces, namely Alberta, British Columbia, Manitoba, Newfoundland, Nova Scotia, Ontario, Prince Edward Island, and Saskatchewan. The present investigation includes only pancreatic cancer cases, and is restricted to data obtained from direct (rather than proxy) interviews. The study population has been described in detail elsewhere (Villeneuve *et al*, 2000). Briefly, between April 1, 1994 and December 31, 1997, the participating provincial registries identified pancreatic cancer cases as early as possible in the registration process, to minimise the loss of subjects from this rapidly fatal disease. All pancreatic cancer cases included in the NECSS were confirmed histologically, and defined according to the WHO's International Classification of Diseases, rubric 157 (WHO, 1985). Like most other studies of pancreatic cancer, the proportion of cases subject to direct interview was low. Among men diagnosed with pancreatic cancer, 30% had died before an interview could be conducted, and consent was not granted by physicians for an additional 15%. Among women, 28% had died before they could be contacted, and the attending physician refused consent to approach patients for an additional 16%. The vast majority of cases were ascertained within 1–3 months of diagnosis; physician consent to contact patients was generally obtained within 1 month, and approximately 70% of questionnaires were returned within 2 months of mailing. Response rates of eligible cases were 55% for men and 56% for women.

The NECSS used frequency matching in selecting the control population to achieve age and gender distributions similar to those of all cancer cases combined. Based on the projected number of incident cancer cases by province, the questionnaires were mailed to 8117 subjects during the 1996 calendar year using the same protocol as for cases. Questionnaires were not returned for 573 controls (7.4%) because of incorrect or changed addresses. Strategies for control selection varied by province, depending on data accessibility. In Prince Edward Island, Nova Scotia, Manitoba, Saskatchewan, and British Columbia, provincial health insurance plans were tapped to obtain a random sample of the provincial population stratified by age and gender. In each of these provinces, more than 95% of residents are covered by public health care plans. Active military personnel and their families as well as indigenous peoples were excluded because they were covered by other plans. In Ontario, Ministry of Finance data were used to derive a stratified random sample, while Newfoundland and Alberta adopted a random digit-dialling method to enrol a population-based sample of controls. A total of 5039 controls were selected to serve as a common control group for all types of cancer. Response rates of 65 and 71% were achieved from the respective male and female control populations.

Questionnaires, with telephone follow-up for clarification when necessary, were mailed to subjects to obtain information on residential and occupational history and other risk factors for cancer. The NECSS questionnaire included questions on smoking history, height, weight, physical activity, and educational attainment.

### Dietary assessment

Food consumption data were obtained via a semi-quantitative food frequency questionnaire (FFQ) developed after two instruments that have been widely validated previously: the short Block questionnaire (Block *et al*, 1990) and the Willett questionnaire (Willett, 1998). Subtle changes were made to the questionnaire items to take into account differences in American and Canadian dietary practices. The FFQ includes questions on 69 different food and beverage items, including the frequency of consumption and the amounts consumed. Participants were asked how often they had consumed these foods per week in the period 2 years prior to interview. Daily FA intakes were determined by summing the FA content of each food and beverage item in the FFQ, based on reference values given in the 1997 Canadian Nutrient File (CNF).

### Statistical analysis

Data were collected on 475 cases (264 men, 211 women) and 4802 controls (2377 men, 2425 women). We excluded subjects with daily energy intake <500 kcal (10 cases and 54 controls) or >5000 kcal (three cases and 27 controls) because such intakes are unrealistic and hence of questionable validity. Finally, a total of 462 cases (258 men, 204 women) and 4721 frequency-matched controls (2331 men, 2390 women) were eligible for analysis.

Food intake among the cases and controls was converted to specific FA intake based on the CNF. To determine the associations between dietary FA intakes and pancreatic cancer risk, the study subjects were divided into four categories according to quartiles of calorie-adjusted FA intake in the control population. Odds ratios (ORs) and associated 95% confidence intervals (CIs) were calculated based on unconditional logistic regression. Risk estimates were adjusted for matching variables (age group and province), lifetime cigarette consumption (0, >0–15, and >15 pack-years), body mass index (BMI) (<25, 25–29.9, and ⩾30 kg m^−2^), educational attainment (years), total fat intake (g day^−1^), and total energy intake. To evaluate effect modification by BMI, age, educational attainment, smoking, and physical activity, the *P*-value for a multiplicative interaction term added to the fully adjusted model was examined – and when it was statistically significant, the analysis was stratified on that variable. Tests for linear trend in the variables included in logistic regression models were performed with scores derived from the median values of categorised variables, and entered into the model as successive integers. All tests of statistical significance were two-sided. Data analysis was performed using SPSS for WINDOWS (release 10.02, 1999; SPSS Inc., Chicago, IL, USA).

## RESULTS

Selected characteristics of the study population are presented in Table 1. The age distributions of cases and controls were somewhat similar although there was a slight excess of younger control subjects, and cases were more likely to consume high amounts of tobacco. Two years prior to the diagnosis of cancer, a tendency towards heightened pancreatic cancer risk with increased BMI was noted, while there were no appreciable differences between cases and controls with respect to physical activity. Cases were more likely to have higher fat intake and greater total energy intake than controls.

The major sources of butyric acid were milk and icecream. Lauric acid, stearic acid, and saturated FAs were derived primarily from butter and cheese. Palmitic acid was mainly supplied by bacon, while oleic acid, linoleic acid, and alpha-linolenic acid were derived from nuts and butter, nuts and chips, and mayonnaise, respectively. Arachidonic acid was from fish and chicken, eicosapentaenoic acid and docosahexaenoic acid were exclusively from fish, while *trans* FAs were from margarine and mayonnaise.

The relationships between lifestyle variables and selected FAs are summarised in Table 2. Age was positively associated with both palmitate and saturated FAs, and inversely associated with oleate and monounsaturated FAs, but not with stearate. BMI was inversely related to palmitate, saturated and monounsaturated FAs, but not to stearate or oleate. Smoking was positively linked with palmitate, stearate, oleate, saturated and monounsaturated FAs. Educational attainment was positively associated with palmitate and inversely associated with stearate, but not with oleate, saturated or monounsaturated FAs. Total fat intake was positively related to stearate, oleate, saturated and monounsaturated FAs, but inversely related to palmitate.

Table 3 presents the ORs and corresponding 95% CIs for pancreatic cancer according to categories of dietary FA intakes. After adjustment for age, province, educational attainment, smoking, BMI, total fat, and total energy intake, a significant inverse association was observed in women between pancreatic cancer risk and palmitate (OR=0.73; 95% CI 0.56–0.96; *P*-trend=0.02), stearate (OR=0.70; 95% CI 0.51–0.94; *P*-trend=0.04), oleate (OR=0.75; 95% CI 0.55–1.02; *P*-trend=0.04), total saturated FAs (OR=0.67; 95% CI 0.50–0.91; *P*-trend=0.01), and monounsaturated FAs (OR=0.72; 95% CI 0.53–0.98; *P*-trend=0.02), when comparing the highest quartile of intake to the lowest.

There was a significant interaction between BMI and stearic acid (*P*=0.04) as well as oleic acid (*P*=0.04), and both total saturated and monounsaturated FAs (*P*=0.05). The risk of pancreatic cancer in relation to intake of stearate, oleate, total saturated and monounsaturated FAs is presented in Table 4 by categories of BMI. Among obese subjects, strong and significant inverse associations were apparent between stearic acid (OR=0.36; 95% CI 0.18–0.70; *P*-trend=0.001), oleic acid (OR=0.36; 95% CI=0.19–0.72; *P*-trend=0.002), saturated FAs (OR=0.35; 95% CI 0.18–0.67; *P*-trend=0.002), and monounsaturated FAs (OR=0.32; 95% CI 0.16–0.61; *P*-trend=0.001) and pancreatic cancer risk, when comparing the highest to the lowest quartile of intakes. There was no evidence of effect modification by age, educational attainment, smoking, and physical activity (*P* for interaction >0.05 in all cases, data not shown).

## DISCUSSION

In this population-based case–control study of specific FAs and pancreatic cancer, we found that palmitic acid, stearic acid, and total saturated FA intakes were associated with a significantly decreased risk. Our findings are in agreement with those of five previous case–control studies that reported statistically significant or borderline decreased risks of pancreatic cancer with higher saturated fat intake, with a relative risk of 0.3 (95% CI 0.1–0.8), 0.3 (95% CI 0.1–1.0), 0.2 (95% CI 0.1–0.2), and 0.9 (*P*-trend=0.028; for men) for intakes in the highest as compared to the lowest quartiles (Baghurst *et al*, 1991; Bueno de Mesquita *et al*, 1991; Zatonski *et al*, 1991; Silverman *et al*, 1998), respectively. A large collaborative population-based case–control of pancreatic cancer comprising 802 cases from five countries in the Surveillance of Environmental Aspects Related to Cancers in Humans (SEARCH) Study found a nonsignificant reduced risk, with an overall OR of 0.8 (95% CI 0.6–1.2) for the highest quartile of saturated fat intake (Howe *et al*, 1992), with significant inverse associations reported in two study centres (Howe and Burch, 1996).

In contrast, three case–control and two cohort studies reported no significant association (Kalapothaki *et al*, 1993; Howe and Burch, 1996; Michaud *et al*, 2003) or an increased risk associated with higher saturated fat intake (Ghadirian *et al*, 1991; Stolzenberg-Solomon *et al*, 2002). The latter investigations reporting an increased risk utilised specific populations such as French Canadians or male smokers who may have particular food habits and dietary patterns. Levels of saturated FA intakes were substantially higher in the case–control by Ghadirian *et al* and in the cohort study by Stolzenberg–Solomon *et al*. The mean saturated fat intake among French-Canadians was 55.2±40.6 g day^−1^ for cases and 42.2±27.0 g day^−1^ for controls compared with 18.2±11.3 g day^−1^ for cases and 20.0±13.7 for controls in our study. The median saturated fat intake among male smokers was 61.8 g day^−1^ for cases and 58.5 g day^−1^ for nonaffected, compared with 16.2 g day^−1^ for cases and 15.3 g day^−1^ for controls in the present investigation; this observation may partly explain the lack of consistency among these studies.

Palmitate and stearate are the most common long-chain saturated FAs. The dietary intake of these two specific was related to 27–30% risk reduction, and a 33% decrease in pancreatic cancer risk with total saturated FA intake. The mechanisms by which these relationships may be explained are areas of current active research. A high fat intake affects pancreatic cancer risk by stimulating cholecystokinin (CCK) release, which, in rodents, increases susceptibility to carcinogens and causes acinar cell hyperplasia, followed by the development of pancreatic carcinomas (Longnecker, 1993; Chu *et al*, 1997). In humans, it has been suggested that duodenal CCK release might differ significantly according to the degree of fat saturation, with unsaturated FAs being stronger stimulants of CCK release than saturated FAs (Beardshall *et al*, 1989). In response to meals containing different fats, CCK release is enhanced by one-, two-, and six-fold for saturated, monounsaturated, and polyunsaturated FAs, respectively. If the hypothesis that the promoting effect of dietary fat on pancreatic carcinogenesis is mediated via CCK is correct, this could partly explain the reduced pancreatic cancer risk associated with both specific and total saturated FAs.

Oleic acid is a major monounsaturated FA. We found a 25% (statistically significant) risk reduction related to oleate intake, with monounsaturated FA intake associated with a 28% decrease in pancreatic cancer risk. One case–control study (Zatonski *et al*, 1991) in Poland found a strong decrease in pancreatic cancer risk (OR=0.10; *P*-trend=0.02) with higher monounsaturated fat intake. Another case–control study (Bueno de Mesquita *et al*, 1990) in the Netherlands reported a 60% nonsignificant decrease in risk among men, but not among women. Other studies have detected no statistically significant association with monounsaturated FAs (Howe *et al*, 1990; Michaud *et al*, 2002).

Few risk factors for pancreatic cancer have been consistently identified. In the present study age, BMI, smoking, educational attainment, and total fat intake were clearly related to palmitate, stearate, oleate, saturated and monounsaturated FAs. We found that adjustments increased the magnitude of the association between these FAs and pancreatic cancer risk. Therefore, it is likely that the factors we included in the model are simultaneously confounding and may be responsible for this effect.

Pancreatic cancer risk reduction in relation to dietary intake of stearic acid, oleic acid, and both total saturated and monounsaturated FAs remained evident among obese subjects. Saturated and monounsaturated FA intakes were associated with strong and significantly decreased pancreatic cancer risk in obese subjects (BMI⩾30), with 65 and 68% risk reductions, respectively. A cohort study of pancreatic cancer indicated that dietary glycaemic load and the glycaemic index are associated with increased risk of pancreatic cancer among women who were overweight, and among obese women with low physical activity (Michaud *et al*, 2002). Obesity is a physiological state that is accompanied by abnormal glucose metabolism and greater insulin resistance. Since the pharmacokinetic properties of both saturated and monounsaturated FAs are not known to depend on BMI, it is likely that obese subjects may be particularly susceptible to the type of FA they consume, likely because of some degree of repression of CCK release. However, the significance of such susceptibility is speculative and remains to be established.

The major strengths of our study included the large number of pancreatic cancer cases, histological diagnosis, and general population sampling of controls. The large sample size permitted subgroup analyses. Histological confirmation of diagnosis reduced the possibility of disease misclassification, while a population-based approach facilitates extrapolation of results to the general population.

Our study is also subject to certain limitations. Since the assessment of dietary exposure was retrospective, recall bias cannot be completely excluded. A prospective cohort approach has several advantages when studying associations between nutritional factors and cancer risk. However, because of the relatively low incidence of pancreatic cancer, the largest cohort to date examining diet and pancreatic cancer risk confirmed only 178 pancreatic cancer cases in 18 years of follow-up (Michaud *et al*, 2003).

We could not adjust OR estimates for the potentially confounding effects of diabetes mellitus and family history of pancreatic cancer, since this information was not collected at baseline. Nevertheless, we expected that diabetes did not confound the association between specific FA intakes and pancreatic cancer risk because of the likelihood that diet represents the initial risk factor for both chronic diseases, rather than lying on the causal pathway. In addition, it has been suggested that diabetes might be one of the early manifestations of pancreatic cancer, and the significance of diabetes is much weaker if cases of recent onset are excluded (WHO/AIRC, 2003). As well, it is unlikely that the confounding effect of family aggregation of pancreatic cancer may explain the significantly inverse associations we found because genetic/familial predisposition is rare (Ghadirian *et al*, 2003). Another limitation is the consequence of early and high case fatality associated with the disease. As in other epidemiological studies of pancreatic cancer, cases died before the questionnaire could be administered were not included in the analysis. However, since there was no discrimination in the selection of study subjects based on their demographic characteristics and lifestyle factors, such as age, smoking or educational attainment, survivors are still representative of the study population. Consequently, bias of this kind, if any, is unlikely to be substantial.

In conclusion, our current analyses suggest that, independently of total energy intake, substituting polyunsaturated FAs with saturated or monounsaturated FAs may reduce pancreatic cancer risk, particularly in subjects who are obese. Further epidemiological studies assessing the role of specific dietary FA intakes in the aetiology of pancreatic cancer are warranted.

## Figures and Tables

**Table 1:** Selected characteristics of the study population (*n*=5183), National Enhanced Cancer Surveillance System, Canada, 1994–1997

	**Cases (*n*=462)**		**Controls (*n*=4721)**	
**Characteristic**	***n***	%	***n***	%
*Age (years)*				
30–34	5	1	186	4
35–39	8	2	264	6
40–44	18	4	333	7
45–49	28	6	448	9
50–54	48	10	426	9
55–59	58	13	486	11
60–64	85	18	701	15
65–69	115	25	914	19
70–74	97	21	963	20

*Cigarette consumption (pack-years)*				
0	144	31	1767	38
>0–15	93	20	1384	29
>15	219	48	1517	32
Missing	6	1	53	1

*BMI 2 years prior to diagnosis (kg m*^−*2*^)				
<25	188	41	2235	47
25–29.9	173	37	1816	39
⩾30	101	22	670	14

*Physical activity (total number of hours week*^−*1*^)				
<21	135	29	1168	25
21–23	228	49	2550	54
⩾23	99	22	1003	21

Daily fat intake^a^ (mean±s.d. in g)	61±35	55±28		
Daily energy intake^a^^b^ (mean±s.d. in kcal)	1862±757	1723±642		

a These differences are significant, *P*<0.001.

b These differences are significant, *P*<0.001.

**Table 2:** Relationships between selected lifestyle factors and calorie-adjusted fatty acid intakes

**Fatty acid**	**Age**	**BMI**	**Smoking**	**Educational attainment**	**Total fat intake**
		**Beta (s.e.)^a^**			
Palmitate	0.25 (0.1)*	−0.96 (0.3)†	0.26 (0.1)†	0.12 (0.4)*	−4.27 (1.4)*
Stearate	−0.11 (0.1)	−0.14 (0.2)	0.28 (0.0)†	−0.49 (0.2)*	85.10 (0.7)†
Oleate	−0.32 (0.1)†	0.25 (0.1)	0.13 (0.0)†	0.04 (0.2)	89.00 (0.6)†
Saturated FAs	0.15 (0.1)*	−0.53 (0.2)*	0.12 (0.0)*	−0.11 (0.2)	82.00 (0.8)†
Monounsaturated FAs	−0.32 (0.1)†	0.21 (0.1)	0.12 (0.0)†	0.04 (0.2)	90.70 (0.6)†

a FA=fatty acid. Parameter estimates (*β* coefficient and standard error in %) from regression with palmitate, stearate, oleate, saturated and monounsaturated FAs as dependent variables and age, BMI, smoking, educational attainment and total fat intake as independent variables.

* *P*<0.05.

† *P*⩽0.001.

**Table 3:** ORs^a^ and 95% CIs for pancreatic cancer associated with dietary fatty acids

	**Quartiles of fatty acid intakes**				
**Fatty acid**	**Q1**	**Q2**	**Q3**	**Q4**	***P*-trend**
*Butyric acid*					
Median (g day^−1^)	0.5	0.5	5	31	
Cases/controls	134/1180	124/1181	92/1181	112/1179	
Multivariate OR (95% CI)	1.00	1.07 (0.81–1.42)	0.78 (0.57–1.05)	0.94 (0.70–1.25)	0.62

*Lauric acid*					
Median (g day^−1^)	0.2	0.3	0.4	1	
Cases/controls	116/1180	113/1180	103/1181	130/1180	
Multivariate OR (95% CI)	1.00	0.99 (0.75–1.32)	0.92 (0.69–1.23)	0.92 (0.70–1.22)	0.56

*Palmitic acid*					
Median (g day^−1^)	7.7	7.7	12	37	
Cases/controls	142/1180	100/1182	107/1179	113/1180	
Multivariate OR (95% CI)	1.00	0.70 (0.53–0.93)	0.74 (0.56–0.97)	0.73 (0.56–0.96)	0.02

*Stearic acid*					
Median±s.d. (g day^−1^)	2.7	3.1	3.9	6.8	
Cases/controls	123/1180	107/1180	111/1181	121/1180	
Multivariate OR (95% CI)	1.00	0.81 (0.61–1.08)	0.82 (0.62–1.09)	0.70 (0.51–0.94)	0.04

*Oleic acid*					
Median (g day^−1^)	13.7	14.6	17.8	28.4	
Cases/controls	121/1180	112/1115	100/1181	125/1180	
Multivariate OR (95% CI)	1.00	0.91 (0.69–1.20)	0.78 (0.59–1.04)	0.75 (0.55–1.02)	0.04

*Linoleic acid*					
Median (g day^−1^)	4.2	4.5	5.6	8.7	
Cases/controls	122/1180	111/1181	108/1180	121/1180	
Multivariate OR (95% CI)	1.00	0.93 (0.70–1.23)	0.90 (0.68–1.19)	0.88 (0.66–1.17)	0.28

*Alpha-linolenic acid*					
Median (g day^−1^)	0.6	0.6	1.1	1.9	
Cases/controls	106/1180	123/1180	115/1181	118/1180	
Multivariate OR (95% CI)	1.00	1.20 (0.90–1.57)	1.10 (0.83–1.47)	0.99 (0.74–1.33)	0.71

*Eicosapentaenoic acid (EPA)*					
Median (g day^−1^)	0.03	0.03	0.04	0.13	
Cases/controls	119/1180	108/1181	122/1180	113/1180	
Multivariate OR (95% CI)	1.00	0.99 (0.75–1.33)	1.16 (0.88–1.54)	1.10 (0.82–1.47)	0.52

*Docosahexaenoic acid (DHA)*					
Median (g day^−1^)	0.02	0.07	0.13	0.37	
Cases/controls	117/1180	105/1182	120/1179	120/1180	
Multivariate OR (95% CI)	1.00	0.96 (0.72–1.28)	1.12 (0.84–1.48)	1.16 (0.876–1.54)	0.30

*Arachidonic acid*					
Median (g day^−1^)	0.02	0.04	0.05	0.08	
Cases/controls	116/1180	109/1180	118/1181	119/1180	
Multivariate OR (95% CI)	1.00	0.96 (0.73–1.28)	1.05 (0.80–1.38)	0.96 (0.73–1.27)	0.85

*ω-3 FAs (EPA+DHA)*					
Median (g day^−1^)	0.02	0.09	0.18	0.50	
Cases/controls	117/1180	107/1181	122/1180	116/1180	
Multivariate OR (95% CI)	1.00	0.97 (0.73–1.30)	1.14 (0.86–1.52)	1.12 (0.84–1.50)	0.40

*Saturated FAs*					
Median (g day^−1^)	11.2	12.5	15.7	28.8	
Cases/controls	127/1180	110/1180	108/1181	117/1180	
Multivariate OR (95% CI)	1.00	0.83 (0.63–1.09)	0.79 (0.60–1.04)	0.67 (0.50–0.91)	0.01

*Monounsaturated FAs*					
Median (g day^−1^)	13.6	15.4	19.0	30.3	
Cases/controls	122/1180	116/1181	101/1180	123/1180	
Multivariate OR (95% CI)	1.00	0.88 (0.67–1.16)	0.76 (0.57–1.02)	0.72 (0.53–0.98)	0.02

*Polyunsaturated FAs*					
Median (g day^−1^)	5.2	5.6	7.1	10.9	
Cases/controls	123/1180	105/1181	117/1180	117/1180	
Multivariate OR (95% CI)	1.00	0.90 (0.68–1.19)	0.97 (0.74–1.28)	0.83 (0.62–1.11)	0.22

*trans-FAs*					
Median (g day^−1^)	0.3	0.6	1.1	2.8	
Cases/controls	97/1180	114/1181	123/1180	128/1180	
Multivariate OR (95% CI)	1.00	1.21 (0.90–1.62)	1.21 (0.90–1.62)	1.08 (0.80–1.44)	0.50

a FA=fatty acid; OR=odds ratio; CI=confidence interval. Odds ratios and 95% confidence intervals from the logistic regression model adjusted for age, province, educational attainment, smoking, BMI, total fat and energy intake.

**Table 4:** ORs^a^ and 95% CIs for pancreatic cancer associated with saturated and monounsaturated fatty acids and body mass index

		**Quartiles of fatty acid intakes**				
**Body mass index**	**Fatty acids**	**Q1**	**Q2**	**Q3**	**Q4**	***P*-trend**
<25	Stearic acid	1.00	0.95 (0.61–1.48)	1.19 (0.78–1.81)	0.71 (0.43–1.17)	0.54
	Oleic acid		0.96 (0.62–1.48)	0.92 (0.60–1.42)	0.76 (0.46–1.25)	0.28
	Saturated FAs		1.12 (0.72–1.73)	1.08 (0.70–1.68)	0.79 (0.49–1.27)	0.44
	Monounsaturated FAs		0.93 (0.61–1.44)	0.91 (0.59–1.41)	0.76 (0.46–1.26)	0.30

25–29.9	Stearic acid	1.00	0.79 (0.50–1.26)	0.81 (0.51–1.29)	0.95 (0.58–1.55)	0.87
	Oleic acid		1.02 (0.64–1.63)	0.88 (0.55–1.42)	1.10 (0.67–1.82)	0.90
	Saturated FAs		0.92 (0.58–1.45)	0.82 (0.52–1.30)	0.84 (0.51–1.37)	0.48
	Monounsaturated FAs		0.93 (0.59–1.47)	0.84 (0.52–1.35)	1.07 (0.64–1.76)	0.97

⩾30	Stearic acid	1.00	0.62 (0.34–1.14)	0.38 (0.20–0.74)	0.36 (0.18–0.70)	0.001
	Oleic acid		0.67 (0.36–1.23)	0.36 (0.18–0.72)	0.36 (0.19–0.72)	0.002
	Saturated FAs		0.40 (0.21–0.76)	0.42 (0.23–0.79)	0.35 (0.18–0.67)	0.002
	Monounsaturated FAs		0.72 (0.39–1.31)	0.37 (0.19–0.73)	0.32 (0.16–0.61)	0.001

a FA=fatty acid. Odds ratios and 95% confidence intervals from the logistic regression model adjusted for age, province, educational attainment, smoking, total fat and energy intake.
